# A guideline proposal for mice preparation and care in ^18^F-FDG PET imaging

**DOI:** 10.1186/s13550-022-00921-y

**Published:** 2022-08-13

**Authors:** F. M. Ribeiro, P. M. M. Correia, A. C. Santos, J. F. C. A. Veloso

**Affiliations:** 1 Institute for Nanostructures, Nanomodelling and Nanofabrication (i3N), Department of Physics, University of Aveiro (DFis-UA), 3810-193 Aveiro, Portugal; 2 Institute for Clinical and Biomedical Research (iCBR), Faculty of Medicine of the University of Coimbra (FMUC), Area of Environment Genetics and Oncobiology (CIMAGO), Center for Innovative Biomedicine and Biotechnology (CIBB), 3000-548 Coimbra, Portugal

**Keywords:** Guideline, Standardization, Small-animal PET imaging, ^18^F-FDG, Mouse handling

## Abstract

The experimental outcomes of small-animal positron emission tomography (PET) imaging with ^18^F-labelled fluorodeoxyglucose (^18^F-FDG) can be particularly compromised by animal preparation and care. Several works intend to improve research reporting and amplify the quality and reliability of published research. Though these works provide valuable information to plan and conduct animal studies, manuscripts describe different methodologies—standardization does not exist. Consequently, the variation in details reported can explain the difference in the experimental results found in the literature. Additionally, the resources and guidelines defining protocols for small-animal imaging are scarce, making it difficult for researchers to obtain and compare accurate and reproducible data. Considering the selection of suitable procedures key to ensure animal welfare and research improvement, this paper aims to prepare the way for a future guideline on mice preparation and care for PET imaging with ^18^F-FDG. For this purpose, a global standard protocol was created based on recommendations and good practices described in relevant literature.

## Introduction

Positron emission tomography (PET) scanners dedicated to small animals emerged in the mid-1990s [1], as animal model-based research of human disease proved to be an essential and extensively used research tool. Molecular imaging enables to measure in vivo molecular pathways and interactions, not only to early identify and describe the biological nature of a disease but also to follow its evolution. Furthermore, it provides direct biological data for the development of potential therapies, as well as for the assessment of their effects [2].

Technical, physical, and physiological factors influence the experimental PET with [^18^F]-fluorodeoxyglucose (FDG) outcomes [3]. For instance, PET scanner design, acquisition time, and image analysis may differ between studies and centres—if the scanner has a high-count rate capability, the activity may be reduced, and PET scan duration increased, thereby keeping ALARA (as low as reasonably achievable) principle in mind. Additionally, many factors such as diet, room temperature, and anaesthesia impact markedly ^18^F-FDG uptake by normal tissues of small animals [4, 5]. In 2019, Mannheim et al*.* [6] detected differences among four preclinical imaging facilities due to different standard PET imaging protocols for ^18^F-FDG, including animal preparation and handling, experimental equipment used, personal performing the PET studies, and image analysis. In the same year, McDougald et al*.* performed a phantom multicentre study recommending acquisition and reconstruction PET/CT protocols, which would improve in vivo measurements independent of scanner design [7]. Besides the incorrect or inappropriate statistical analysis of results or the insufficient sample sizes pointed out by Prinz et al. [8] as one of the reasons for the lack of reproducibility, the absence of a global standard protocol limits repeatability, reproducibility, and reliability of data.

Stout et al*.* [9] proposed a complete report of materials and methods to assist a manuscript submission, while Mannheim et al*.* [10] published a review addressing the current status and prospects of small animal imaging standards, including PET. Furthermore, a checklist of recommendations to improve the reporting of research involving animals, the ARRIVE (Animal Research: Reporting of In Vivo Experiments) guidelines 2.0. were recently launched [11]. Although these works provide valuable information to plan and conduct animal studies, ensuring reproducibility through a detailed report, the authors still mentioned the need to develop specific animal preparation and care protocols for a particular biomarker. This issue had already been emphasized by Chalmers and Glasziou [12] while examining the causes and degree of waste in producing and reporting research evidence. The authors concluded that a loss of about 50% in the quality of research design and methods, adequacy of publication practices, and quality of reports of research would lead to a loss greater than 85% [12]. Tackling this problem has become an international effort to ensure that data from in vivo studies provide reliable results to the scientific knowledge [13–15].

Therefore, the present work aims to propose a guideline to provide a minimum standard for the preparation and care of mice for PET imaging with ^18^F-FDG. The global standard protocol focuses on the practical steps that can be controlled and suggested for broad adoption. For this purpose, recommendations and good practices described in relevant literature were assessed.

### Principle

PET is a non-invasive imaging modality that quantifies the 3-dimensional distribution of a retained positron-emitting compound as a function of time. Thus, allowing to measure physiological, biochemical, and pharmacokinetic parameters such as: blood flow, glucose and oxygen metabolism, pre/postsynaptic receptor density and affinity, neurotransmitter release, enzymatic activity, drug distribution and uptake, gene expression, among others [16]. Hence, PET technology is a standard indication in oncology, neurology, and cardiology [17, 18]. Furthermore, PET imaging enables to conduct longitudinal studies, thus, each animal can be its own control, eliminating inter-individual variability. Consequently, there is a considerable reduction of the number of animals (complying with the 3Rs principles), and cost associated with the experiments, enabling a quite fast availability of results.

The most used tracer is the glucose analogue, FDG. The uptake of ^18^F-FDG reflects the regional glucose consumption, allowing to study altered metabolic states associated with a wide variety of brain and heart disorders and diseases, as well as in oncology [2]. ^18^F-FDG gained relevance quite fast regarding the detection and biological characterization of tumour tissue [19], monitoring of tumour progression and transformation [20], treatment planning, and follow-up [21, 22]. Additionally, ^18^F-FDG is commonly used as a gold standard when assessing other imaging probes [23–25].

Before starting the experiment, perform cross-calibration of the clocks used and between PET scanner and dose calibrator. Osborne et al*.* [26] outlined a set of quality control guidelines for small animal imaging, encompassing expert-driven suggestions and alternatives for laboratories that do not have access to specialized phantoms for testing. Consult the Directive 2010/63/EU on the protection of animals used for scientific purposes. This Directive sets out legal requirements to implement the 3Rs principles of replacement, reduction, and refinement, harmonizing animal research legislation throughout the European Union, thus ensuring high standards of animal welfare and scientific research.

It is of utmost importance to select the appropriate procedures for each study and to ensure that the parameters set during the experiment remain constant. In ^18^F-FDG PET imaging, animal preparation and care can compromise the research results by possibly masking glucose metabolic activity of targets due to a high glucose consumption in different background tissues. Hence, the main purpose is to maintain and optimize the ^18^F-FDG in the target structures. In the following, a generally applicable protocol is outlined.

## Mouse preparation and care prior to ^18^F-FDG PET imaging

### Animal handling

During a PET study, where the interaction between the animals and the person performing the PET imaging is often of short duration and low frequency, mice should be handled indirectly in a home cage tunnel. Gouveia and Hurst [27] investigated the duration and frequency of handling required for effective familiarization with non-aversive handling methods, namely tunnel and cup handling, compared to tail handling. The findings demonstrated that 2 s of handling during cage cleaning were sufficient to familiarize mice with tunnel handling, while brief but more frequent handling was needed for cup handling. Even after experiencing complete immobilization by scruff restraint, mice familiarized with tunnel handling continue to interact with the handler, while maintaining the level of anxiety reduced. As for mice picked up by the tail, even when handled briefly and infrequently, they showed strong aversion and anxiety.

### Housing

The minimum cage height and floor size should be 12 cm and 330 cm^2^, respectively, for one individual housed adult, according to the Directive 2010/63/EU. Isolate the mouse in a clean cage prior to ^18^F-FDG administration, removing food but always keeping water available ad libitum*.* In the work of Cao et al*.* [28], fasting promoted the general activity of the animal due to food search and, consequently, ingestion of litter or faeces (coprophagy), stress, and aggression. As prevention, remove bedding material during the fasting period, as Prior et al*.* [29] advocate, but maintain environmental enrichment to enhance the physical and psychological well-being of the mouse, fulfilling its need to find shelter. If relevant, consult the work of Baumans and Loo [30], who studied the possibilities of environmental refinement in terms of benefit to the animal.

### Temperature

The metabolic rate of rodents is minimal and theoretically equal to the basal metabolism rate in the so-called thermoneutral zone (26–34 °C) [31]. In this range, body temperature is essentially regulated by conduction and heat convection [32]. Due to the body surface area to body volume ratios and metabolic rate (practically tenfold higher than larger mammalian species), the thermal mass to keep the body warm is also reduced, being associated with the high rodent metabolism which makes them more susceptible to hypothermia [33]. Therefore, set the room temperature between 18 and 26 °C [32, 34] and keep the body temperature of the mouse between 36.5 and 38 ºC employing heating devices (electrical or microwavable heating pads, warm air devices, hot water bags or bottles, circulation of hot water or hair blankets, lamps).

While planning your PET studies, consider that the body temperature peak occurs at the beginning of the scoto-phase, in contrast with the lowest temperature value that is observed at the middle of the photo-phase [35, 36]. Hence, fasting and anaesthesia effects on murine temperature may result in hypothermia. After 7–8 h of fasting, the metabolic rate may be suppressed up to 30% regarding the basal metabolism rate, accompanied by a temperature drop to 15 °C [37], and 15–20 min of general anaesthesia induces a decrease in temperature in the centre of the body of approximately 4–10 °C [38]. The possible consequences of hypothermia are cardiac arrhythmias, decreased protection against infections, long post-anaesthetic recovery time, a decrease in the minimum alveolar concentration values (increased toxicity potential) [39], and death within minutes (5–6 min at 20 ºC) [40]. If measurements or injections are performed on/into the tail vein, temperature monitoring is even more relevant since the blood flow is sensitive to temperature due to thermoregulation through the tail.

### Blood glucose level and diet conditions

The circadian rhythm controls several physiological factors such as glucose metabolism: glucose concentration increases at the beginning of the photo-phase (presenting a peak in its middle) and decreases with the beginning of the scoto-phase, remaining at a base level [41]—Froy [42] summarized findings linking the circadian rhythm and several physiological parameters, including glucose metabolism. Whenever possible, schedule your PET experiments to the same time of day to minimize this variable.

Fasting is required to decrease blood glucose level, avoiding competition between glucose analogue (FDG) and endogenous glucose for its transporters to ensure uptake by the target. For more detail, Dolat and Sazgarnia [43] presented a review to compare the results of some clinical studies regarding the effects of fasting on the function of important organs; and Jensen et al*.* [41] presented a review about the effects of fasting in mice, providing evidence for fasting-induced changes in hormone balance, body weight, metabolism, hepatic enzymes, cardiovascular parameters, body temperature, and toxicology responses.

Concerning the optimal duration of fasting, Fuger et al*.* [4] kept mice fasting for 8 to 12 h and Woo et al*.* [44] for 20 h. Lee et al*.* [45] and Deleye et al*.* [46], both noticed that metabolic effects were attenuated after 20 h of fasting. According to the review of Rowland [47] on the important physiological effects of deprivation or restricted access to either food or fluids in common laboratory animals, a 24 h fasting represents an acceptable weight loss (< 10%), since they are physiologically prepared to tolerate acute suppression of food for periods in multiples of 24 h of duration and periodicity. However, fasting duration should be as short as possible when mice need to be repeatedly scanned within a short period, as it results in substantial weight loss [46]. A reasonable fasting period was found by Siikanen et al*.* [48]: 4 h was considered sufficient to achieve a stable blood glucose level, being near the 5–6 h advised for a better comparison to humans by Jensen et al*.* [41].

Prior to administering ^18^F-FDG, measure the blood glucose level (106–278 mg/dL [49])—a Glucometer or a similar device can be used for this purpose. Collect a 3 µl blood sample [50] using a syringe (0.6–1.0 mm needle) or a puncture in the lateral or ventral vein of the tail [32, 49]. If cannulation for ^18^F-FDG administration is unsuccessful, blood may be used from this puncture, and ^18^F-FDG should be injected on the opposite side [50]. Moreover, heat the site with hot water, for example, to induce vasodilation.

## Anaesthesia

Inhalational anaesthesia is the method of choice and the commonly used inhalable anaesthetic is isoflurane. For isoflurane, the carrier gas is usually 100% oxygen, mitigating the effects of respiratory depression and hypoxia [39]. For more detail, the work of Gargiulo et al*.* [51] reviews the existing literature on anaesthetic protocols adopted in mice for molecular imaging studies and a review article that discusses the science of mouse anaesthesia was recently published by Navarro et al*.* [39].

In general, the procedure for inducing anaesthesia in mice involves the following steps:

- Apply sterile unmedicated eye drops or ointment to prevent corneal damage and desiccation.
- Place the mouse in the induction chamber where it is initially anaesthetized and latch the lid.
- Set the vaporizer concentration between 4–5% + 0.8–1 L/min [51] until the mouse becomes immobile.
- Reduce the vaporizer setting to the appropriate level (1–3% + 0.8–1 L/min [51]) for a stable heart rate and blood pressure.
- The mouse should remain in the induction chamber for 3 to 5 min after initial anaesthetic induction before moving it to the PET instrument.
- Once the mouse is ready, transfer it from the induction chamber onto the bed of the PET camera with a breathing device and mask that supplies a constant flow of the anaesthetic gas, keeping the mouse safely anaesthetized during PET scanning.
- Note: if the animal has subclinical respiratory disease, resulting in hypoventilation and mild respiratory compromise, in the book entitled *Biology and Medicine of Rabbits and Rodents*, Harkness et al*.* [31] advised pre-oxygenation with 100% oxygen for 3 to 5 min prior to anaesthesia induction.

## ^18^F-FDG administration

First, make sure that clocks of the dose calibrator and scanner are synchronized (official local time within 1 min) [52]. Then, maintain the mouse warm for a normal metabolism, reducing interscapular brown fat uptake. For a fast systemic product biodistribution, apply intravenous bolus as administration route using indwelling cannulation to avoid partial paravenous injection (with local ^18^F-FDG retention in surrounding tissues), which is a common error due to the small size and fragility of murine tail veins [32, 34].

The volume of ^18^F-FDG administered should be less than 10% of the total blood volume [17]. Table 1 shows the recommended intravenous administration volume (*V*, mL/kg), maximum volume (*V*_Max_, mL), and needle gauge (G) [32]. Due to the small total blood volume of adult mice (1.6–3.2 mL [31]), the volume of ^18^F-FDG that can be administered is limited. The total activity contained in that volume must be concentrated to obtain an acceptable signal [34]. The activity value depends mainly on the performance of the PET system (sensitivity or enhanced technology), i.e. the minimum recommended ^18^F-FDG activity and PET acquisition duration must be adjusted to obtain a quality image within acceptable limits. One may decide to increase the activity or, preferably, the duration of the acquisition. In all cases, the administered activity, within the field-of-view, should not exceed the peak count rate capability of the scanner in use. Here are some examples of ^18^F-FDG activities applied in mice: Inveon (Siemens, 2009)—9 MBq [53], PETbox4 (UCLA, 2013)—1.5 MBq [54], ClairvivoPET (Shimadzu, 2016)—5.8 MBq [55], IRIS (2017)—7.5 MBq [56], MADPET4 (Bruker, 2017)—11.5 MBq [57], β-CUBE (Molecubes, 2018)—6.5 MBq [58], G8 (Sofie Biosciences, 2018)—1.96 MBq [59], PKU-PET-II (Peking University, 2018)—48 MBq [60], Xtrim-PET (Parto Negar Persia, 2019)—8 MBq [61], nanoScan (Mediso, 2021)—14 MBq [62], and easyPET.3D (RI-TE, 2021) 7.5 MBq [63].

**Table 1:** Good practice volume (*V*, mL/kg), possible maximum volume (*V*_Max_, mL), and recommended needle size (*G*) for intravenous injection in mice

*V* (mL/kg)	*V*_Max_ (mL)	*G*
5 (bolus) 25 (slow)	0.125	25–28

After preparing the syringe, measure and record its activity, as well as its volume and time of measurement. Insert a short catheter (25–28 G needle) filled with heparinized saline right through the skin and into the lateral tail vein—excellent lighting would be helpful, and a light localized heating (warm water, lamp, or heating pad) simplifies the procedure. Advance the tip a couple of millimetres. At that point, there should be no resistance felt while administering ^18^F-FDG, if the placement has been successful. In the end, record the time of ^18^F-FDG administration, route, and site. Record also the residual activity measured in the syringe and catheter, including the time of measurement, to correct the injected dose, especially if the material is left in place during scanning, as it may lead to errors in quantification given the small volume [3].

## During ^18^F-FDG PET imaging

Start positioning the mouse with a slight inclination and the head above the tail, enabling maximal costal movement and avoiding thoracic compression, as suggested by Balaban and Hampshire [33] in their review of the challenges of small animal non-invasive imaging. Then, check if the target is included in the field-of-view. Prevent the animal's eyes from being directly in contact with heat, inhalational anaesthetic agent, or any surface, and prevent as well short- or long-term adverse effects by adjusting the depth of anaesthesia or supportive care, maintaining the physiological state of the mouse as close as possible to normal, to reduce pain and stress and predict possible complications:

- Visual monitoring includes watching the respiration (classifies the character of breathing), the colour of mucous membrane and skin (oxygenation state: blue—poor oxygenation; pale—poor blood perfusion), and the general behaviour of the mouse [3].
- Electronic equipment available for small animal monitoring include rectal probe (body temperature, 36.5–38 °C), electrocardiogram (heart function and rate, 350–700 beats/min), small pneumatic pillow (respiration rate, 80–220 breaths/min), clip-on-sensors (pulse oximeter, 1.63–2.17 mL/g/h), cuff sensor (blood pressure, systolic: 133–160 mmHg; diastolic: 102–110 mmHg) [31, 32, 49].

## Mouse care after ^18^F-FDG PET imaging

Set previously the room temperature between 18 and 26 ºC and prepare a cage stocked with paper and nesting material. During recovery, isolate the animal—if it is not fully awake, it may injure other animals in the cage—and maintain fluid supply ad libitum, as well as monitoring of the vital signs until the mouse is fully recovered. Lastly, disinfect the bed of the PET device with a suitable solution or dispose of the protective material, between groups of animals in the same health condition or individual animals, to prevent disease transmission and possible radioactive contamination due to urine loss during the exam.

## Procedures for specific indications

### Oncology

The time of day the PET experiments are performed at is especially important in oncological treatments, as ^18^F-FDG uptake may vary due to chronotolerance and radiotolerance. Several studies demonstrate this dependence of response to medication and radiation treatments on the time of the day: Sephton et al*.* [64] reported an earlier mortality for metastatic breast cancer patients with an abnormal cortisol rhythm, proving the importance of diurnal rhythm changes to survival; Lévi et al. [65, 66] discussed the importance of timing in cancer therapy, considering the circadian rhythm responsible for predictable changes in the tolerance and efficacy of anticancer agents, as well as in tumour induction or development; the increase in chemotherapy tolerance through chronomodulated chemotherapy in recurrent and metastatic head and neck squamous cell carcinoma was also demonstrated by Chen et al*.* [67]. Therefore, try to perform the PET studies at the same time of the day or reverse the day/night cycle by changing the light sequence in the *vivarium*.

During ^18^F-FDG administration and uptake period, keep mice under isoflurane anaesthesia to markedly decrease the background (brown fat and skeletal muscle), while the heart and blood clearance (liver and kidneys) signals are increased. This recommendation agrees with the protocol for imaging tumour xenografts with ^18^F-FDG by Fueger et al*.* [4] and the recent data from Pattison et al*.* [68], suggesting that isoflurane may improve biodistribution of ^18^F-FDG for tumour imaging.

According to the guidelines for the welfare and use of animals in cancer research, imaging sessions should be limited to 2–3 h in a 24 h period and do not exceed five sessions in a 1–2-week period with no more than one session/day [65].

### Neurology

As previously mentioned, fasting is required in ^18^F-FDG PET imaging in mice. For neurological purposes, it is particularly important given that the cerebral ^18^F-FDG uptake varies inversely with blood glucose level (i.e. higher in fasting mice), as Wong et al*.* [5] concluded while studying the effects of dietary condition (fasting *versus* non-fasting) and blood glucose level on the kinetics and uptake of ^18^F-FDG in mice using intraperitoneal and intravenous routes. Simultaneously, they verified that the cerebral glucose metabolic rate does not differ considerably under different dietary states and administration routes. In agreement, Deleye et al*.* [46] observed a high brain uptake even after 20 h of fasting, recommending corrections for blood glucose levels to attenuate the effect of different fasting durations in brain ^18^F-FDG PET imaging.

Once more, schedule your PET experiments in order to favour target uptake. For instance, Krueger et al*.* [69] studied the effect of the circadian rhythm on ^18^F-FDG uptake in human tumour xenografts, every 4 h over a period from 8 AM to 8 PM. Their results revealed that ^18^F-FDG uptake in the tumour models analysed, and other organs (muscle, heart, and liver) remained stable throughout the day, while the circadian rhythm changed the uptake in the brain from noon to 4 PM.

Toyama et al. [70] proved that anaesthesia reduces neural activity and metabolism inducing a significant high standard deviation of binding potential. However, Bascuñana et al*.* [71] showed that conscious mice during ^18^F-FDG uptake had similar values of whole brain compared to mice anaesthetized with isoflurane, whereas the cortex and cerebellum uptakes were higher for conscious and unconscious mice, respectively. Moreover, Matsumura et al*.* [72] demonstrated that ^18^F-FDG uptake was almost the same as that in conscious state (no anaesthesia) when anaesthesia was induced 40 min after ^18^F-FDG administration. Hence, the ^18^F-FDG administration and uptake should be conscious over 40 min followed by unconscious PET imaging.

Concerning the administration route, intravenous infusion over approximately 5 min instead of a bolus injection showed an optimal accuracy of the initial portion of the blood curve for mouse brain kinetic modelling and allowed for longer blood sampling intervals as stated by Alf et al*.* and Vanhove et al*.* [34, 73]. The work of Mizuma et al*.* [74] established an in vivo brain PET imaging method in conscious mice to assess physiological neural functions, reporting a significant brain ^18^F-FDG uptake increase in mice receiving automatic injection via an indwelling catheter in the tail vein. Besides, the incidence of adverse effects (cardiovascular failure, for example) is decreased through a slow rate of injection [34].

### Cardiology

In studies concerning thoracic malignancies, with or without myocardial involvement, fasting followed by high-fat diet can be advantageous for a greater suppression of physiological myocardial ^18^F-FDG uptake, as Langah et al*.* [75] showed by comparing the effects of four preparation protocols in rodents. Thus, the dietary conditions are again relevant to consider, as example, it was useful to detect inflammatory lesions in the early stage of cardiac sarcoidosis, in the work of Okumura et al*.* [76]. This prerequisite also induces a decrease in insulin levels. Consequently, glucose uptake in the background tissues is reduced enhancing ^18^F-FDG uptake in the target. The previously mentioned work of Wong et al*.* [5] showed that myocardium and skeletal muscle ^18^F-FDG uptake constants are highly affected by dietary conditions (lower in fasting mice) but not by blood glucose level. Similarly, Kreissl et al*.* [77] found a low glucose metabolic rate in the myocardium and skeletal muscle of fasted mice but not in the brain, while evaluating the effect of insulin stimulation and dietary changes on brain, myocardium, and skeletal muscle ^18^F-FDG kinetics and uptake in mice. In the same work, insulin injection increased the ^18^F-FDG uptake rate constant for myocardium (in non-fasted mice) and skeletal muscle (independently of the dietary state). However, in the brain, the parameter measured was not impacted by fasting or administration of insulin.

Like in brain, use conscious ^18^F-FDG administration and uptake over 40 min followed by unconscious PET scanning, since isoflurane alters myocardial glucose metabolism [4, 70] masking any effect of heparin or fasting. Thackeray et al*.* [78] tested suitable approaches for suppression of cardiomyocyte uptake in mouse models, confirming that the effect of isoflurane can be minimized by conscious ^18^F-FDG administration and uptake over 40 min prior to anaesthesia induction.

### Dynamic studies

Unconscious ^18^F-FDG administration and uptake is a prerequisite for performing dynamic PET studies, i.e. the mouse is injected when placed inside the imaging instrument and scanned continuously during a certain time to identify changes in ^18^F-FDG biodistribution. These scans are required to measure the so-called arterial input function along with tissue time activity curve for kinetic modelling of PET data. In 2005, Laforest et al*.* reviewed several techniques for measurement of the blood input function in rodents and Meyer et al*.* evaluated a standardized arterial input function method in mice [79, 80]. Kinetic modelling with PET aims to estimate physiological parameters by extracting transfer rate constants, volumes of distribution, or binding potentials from the radiopharmaceutical to the target. Consult the publication of Amirrashedi et al*.* [81] for current trends in preclinical PET imaging, including the key factors to consider for kinetic modelling.

### Final considerations

Technical, physical, and physiological parameters can affect the outcomes obtained from preclinical imaging techniques. Therefore, standardization of experimental procedures is essential to achieve repeatable, reproducible, and reliable data. Animal preparation and care particularly impact the recorded ^18^F-FDG PET data. Current publications concerning the research report improvement and the quality and reliability of published research amplification. Still, none defines a global standard protocol for preclinical ^18^F-FDG PET imaging procedures. The present overview represents a key milestone in the effort to acquire ^18^F-FDG PET more robust data with less variability, enabling the use of fewer mice and ensuring high standards of animal welfare, thus complying with the 3Rs policy. This guideline focuses on the practical steps for mice preparation and care, which can be controlled and suggested for broad adoption prior, during and after ^18^F-FDG PET imaging. Overall, researchers are encouraged to follow the recommendations outlined herein during the planning and performing of their studies, assuring savings in cost and time, as better results.

## Data Availability

Not applicable.

## References

[1] Cherry SR, Gambhir SS (2001). Use of positron emission tomography in animal research. ILAR J.

[2] Phelps ME (2000). Positron emission tomography provides molecular imaging of biological processes. Proc Natl Acad Sci USA.

[3] Kuntner C, Stout D (2014). Quantitative preclinical PET imaging: opportunities and challenges. Front Phys.

[4] Fueger BJ (2006). Impact of animal handling on the results of 18F-FDG PET studies in mice. J Nucl Med.

[5] Wong K-P, Sha W, Zhang X, Huang S-C (2011). Effects of administration route, dietary condition, and blood glucose level on kinetics and uptake of 18F-FDG in mice. J Nucl Med.

[6] Mannheim JG (2019). Reproducibility and comparability of preclinical PET imaging data: a multicenter small-animal PET study. J Nucl Med.

[7] McDougald W (2020). Standardization of preclinical PET/CT imaging to improve quantitative accuracy, precision and reproducibility: a multi-center study. J Nucl Med.

[8] Prinz F, Schlange T, Asadullah K (2011). Believe it or not: How much can we rely on published data on potential drug targets?. Nat Rev Drug Discov.

[9] Stout D (2013). Guidance for methods descriptions used in preclinical imaging papers. Mol Imaging.

[10] Mannheim JG (2018). Standardization of small animal imaging—current status and future prospects. Mol Imaging Biol.

[11] NC3Rs, “ARRIVE guidelines,” New ARRIVE guidelines 2.0 release, 2020. https://arriveguidelines.org. Accessed Nov. 18, 2020.

[12] Chalmers I, Glasziou P (2009). Avoidable waste in the production and reporting of research evidence. Lancet.

[13] Macleod M (2014). Biomedical research: increasing value, reducing waste. Lancet.

[14] Ioannidis J (2005). Why most published research findings are false. PLoS Med.

[15] Chalmers I (2014). How to increase value and reduce waste when research priorities are set. Lancet.

[16] Lammertsma AA. Role of human and animal PET studies in drug development. Int Cong Ser 2004;1265(C):3–11. 10.1016/j.ics.2004.03.026.

[17] Yao R, Lecomte R, Crawford ES (2012). Small-ANIMAL PET: What is it, and why do we need it?. J Nucl Med Technol.

[18] Bouter C, Bouter Y. 18F-FDG-PET in mouse models of Alzheimer’s disease. Front Med (Lausanne) 2019;6:71. 10.3389/fmed.2019.00071. 10.3389/fmed.2019.00071 PMC6482246 31058151

[19] Dearling J (2004). Analysis of the regional uptake of radiolabeled deoxyglucose analogs in human tumor xenografts. J Nucl Med.

[20] Abbey CK (2004). In vivo positron-emission tomography imaging of progression and transformation in a mouse model of mammary neoplasia. Proc Natl Acad Sci USA.

[21] Bjurberg M, Kjellén E, Ohlsson T, Ridderheim M, Brun E (2007). FDG-PET in cervical cancer: Staging, re-staging and follow-up. Acta Obstet Gynecol Scand.

[22] Adam JA (2021). EANM/SNMMI practice guideline for [^18^F]FDG PET/CT external beam radiotherapy treatment planning in uterine cervical cancer v1.0. Eur J Nucl Med Mol Imaging.

[23] Aliaga A (2004). Breast cancer models to study the expression of estrogen receptors with small animal PET imaging. Nucl Med Biol.

[24] Rau FC (2002). O-(2-[18F]fluoroethyl)-L-tyrosine (FET): A tracer for differentiation of tumour from inflammation in murine lymph nodes. Eur J Nucl Med Mol Imaging.

[25] Zanzonico P (2004). Iodine-124-labeled iodo-azomycin-galactoside imaging of tumor hypoxia in mice with serial microPET scanning. Eur J Nucl Med Mol Imaging.

[26] Osborne DR, Kuntner C, Berr S, Stout D (2017). Guidance for efficient small animal imaging quality control. Mol Imaging Biol.

[27] Gouveia K, Hurst JL (2019). Improving the practicality of using non-aversive handling methods to reduce background stress and anxiety in laboratory mice. Sci Rep.

[28] Cao J, Zhang LN, Zhao ZJ (2009). Trade-off between energy budget, thermogenesis and behavior in Swiss mice under stochastic food deprivation. J Therm Biol.

[29] Prior H, Ewart L, Bright J, Valentin JP (2012). Refinement of the charcoal meal study by reduction of the fasting period. Altern Lab Anim.

[30] Baumans V, van Loo PLP (2013). How to improve housing conditions of laboratory animals: The possibilities of environmental refinement. Vet J.

[31] Harkness JE, Turner PV, VandeWoude S, Wheler CL. Biology and medicine of rabbits and rodents, 5th ed. Blackwell, 2010.

[32] Hubrecht R, Kirkwood J. The UFAW handbook on the care and management of laboratory and other research animals, 8th ed. Wiley-Blackwell, 2010. 10.1002/9781444318777.

[33] Balaban RS, Hampshire VA (2001). Challenges in small animal noninvasive imaging. ILAR J.

[34] Vanhove C, Bankstahl JP, Krämer SD, Visser E, Belcari N, Vandenberghe S (2015). Accurate molecular imaging of small animals taking into account animal models, handling, anaesthesia, quality control and imaging system performance. EJNMMI Phys.

[35] Szentirmai É, Kapás L, Sun Y, Smith RG, Krueger JM (2010). Restricted feeding-induced sleep, activity, and body temperature changes in normal and preproghrelin-deficient mice. Am J Physiol Regul Integr Comp Physiol.

[36] Ms C, Lynch C (1981). Circadian variation of strain differences in body temperature and activity in mice. Physiol Behav.

[37] Swoap SJ, Gutilla MJ, Liles LC, Smith RO, Weinshenker D (2006). The full expression of fasting-induced torpor requires β3-adrenergic receptor signaling. J Neurosci.

[38] Taylor DK (2007). Study of two devices used to maintain normothermia in rats and mice during general anesthesia. J Am Assoc Lab Anim Sci JAALAS.

[39] Navarro KL, Huss M, Smith JC, Sharp P, Marx JO, Pacharinsak C (2021). Mouse anesthesia: the art and science. ILAR J.

[40] Suckow C, Kuntner C, Chow P, Silverman R, Chatziioannou A, Stout D (2009). Multimodality rodent imaging chambers for use under barrier conditions with gas anesthesia. Mol Imaging Biol.

[41] Jensen TL, Kiersgaard MK, Sørensen DB, Mikkelsen LF (2013). Fasting of mice: a review. Lab Anim.

[42] Froy O (2007). The relationship between nutrition and circadian rhythms in mammals. Front Neuroendocrinol.

[43] Dolat E, Sazgarnia A (2014). The effect of fasting on positron emission tomography (PET) imaging: a narrative review photodynamic therapy (PDT) view project hyperspectral imaging for monitoring of food process view project. J Fasting Health.

[44] Woo SK (2008). Anesthesia condition for 18F-FDG imaging of lung metastasis tumors using small animal PET. Nucl Med Biol.

[45] Lee K (2005). Effects of anesthetic agents and fasting duration on 18F-FDG biodistribution and insulin levels in tumor-bearing mice. J Nucl Med.

[46] Deleye S (2016). The effects of physiological and methodological determinants on 18F-FDG mouse brain imaging exemplified in a double transgenic Alzheimer model. Mol Imaging.

[47] Rowland N (2007). Food or fluid restriction in common laboratory animals: balancing welfare considerations with scientific inquiry. Comp Med.

[48] Siikanen J (2015). An anesthetic method compatible with 18 F-FDG-PET studies in mice. Am J Nucl Med Mol Imaging.

[49] Suckow MA, Danneman PJ, Brayton C. The laboratory mouse. SRS Press;2001.

[50] Dandekar M, Tseng JR, Gambhir SS (2007). Reproducibility of 18F-FDG microPET studies in mouse tumor xenografts. J Nucl Med.

[51] Gargiulo S (2012). Mice anesthesia, analgesia, and care, part i: anesthetic considerations in preclinical research. ILAR J.

[52] Boellaard R (2010). FDG PET and PET/CT: EANM procedure guidelines for tumour PET imaging: Version 1.0. Eur J Nucl Med Mol Imaging.

[53] Bao Q, Newport D, Chen M, Stout DB, Chatziioannou AF (2009). Performance evaluation of the inveon dedicated PET preclinical tomograph based on the NEMA-NU4 standards. J Nucl Med.

[54] Gu Z (2013). NEMA NU-4 performance evaluation of PETbox4, a high sensitivity dedicated PET preclinical tomograph. Phys Med Biol.

[55] Sato K (2016). Performance evaluation of the small-animal PET scanner ClairvivoPET using NEMA NU 4–2008 Standards. Phys Med Biol.

[56] Belcari N (2017). NEMA NU-4 performance evaluation of the IRIS PET/CT preclinical scanner. IEEE Trans Radiat Plasma Med Sci.

[57] Omidvari N (2017). PET performance evaluation of MADPET4: A small animal PET insert for a 7 T MRI scanner. Phys Med Biol.

[58] Krishnamoorthy S, Blankemeyer E, Mollet P, Surti S, van Holen R, Karp JS (2018). Performance evaluation of the MOLECUBES β-CUBE - A high spatial resolution and high sensitivity small animal PET scanner utilizing monolithic LYSO scintillation detectors. Phys Med Biol.

[59] Gu Z (2019). Performance evaluation of G8, a high-sensitivity benchtop preclinical PET/CT tomograph. J Nucl Med.

[60] Xie Z (2018). PKU-PET-II: a novel SiPM-based PET imaging system for small animals. Nucl Instrum Methods Phys Res Sect A Accel Spectrom Detect Assoc Equip.

[61] Amirrashedi M (2019). NEMA NU-4 2008 performance evaluation of Xtrim-PET: a prototype SiPM-based preclinical scanner. Med Phys.

[62] Chomet M (2021). Performance of nanoScan PET/CT and PET/MR for quantitative imaging of 18F and 89Zr as compared with ex vivo biodistribution in tumor-bearing mice. EJNMMI Res.

[63] Nicolucci C (2021). Single low dose of cocaine-structural brain injury without metabolic and behavioral changes. Front Neurosci.

[64] Sephton SE, Sapolsky RM, Kraemer HC, Spiegel D (2000). Diurnal cortisol rhythm as a predictor of breast cancer survival. J Natl Cancer Inst.

[65] Lévi F (2006). Chronotherapeutics: the relevance of timing in cancer therapy. Cancer Causes Control.

[66] Levi F, Okyar A, Dulong S, Innominato PF, Clairambault J (2010). Circadian timing in cancer treatments. Annu Rev Pharmacol Toxicol.

[67] Chen D, Cheng J, Yang K, Ma Y, Yang F (2013). Retrospective analysis of chronomodulated chemotherapy versus conventional chemotherapy with paclitaxel, carboplatin, and 5-fluorouracil in patients with recurrent and/or metastatic head and neck squamous cell carcinoma. OncoTargets Ther.

[68] Pattison DA, MacFarlane LL, Callahan J, Kane EL, Akhurst T, Hicks RJ (2019). Personalised insulin calculator enables safe and effective correction of hyperglycaemia prior to FDG PET/CT. EJNMMI Res.

[69] Krueger MA, Calaminus C, Schmitt J, Pichler BJ (2020). Circadian rhythm impacts preclinical FDG-PET quantification in the brain, but not in xenograft tumors. Sci Rep.

[70] Toyama H (2004). Evaluation of anesthesia effects on [18F]FDG uptake in mouse brain and heart using small animal PET. Nucl Med Biol.

[71] Bascuñana P, Thackeray JT, Bankstahl M, Bengel FM, Bankstahl JP (2019). Anesthesia and preconditioning induced changes in mouse brain [18F] FDG uptake and kinetics. Mol Imaging Biol.

[72] Matsumura A (2003). Assessment of microPET performance in analyzing the rat brain under different types of anesthesia: Comparison between quantitative data obtained with microPET and ex vivo autoradiography. Neuroimage.

[73] Alf MF, Martić-Kehl MI, Schibli R, Krämer SD (2013). FDG kinetic modeling in small rodent brain PET: Optimization of data acquisition and analysis. EJNMMI Res.

[74] Mizuma H, Shukuri M, Hayashi T, Watanabe Y, Onoe H (2010). Establishment of in vivo brain imaging method in conscious mice. J Nucl Med.

[75] Langah RAK, Spicer KM, Chang R, Rosol M (2012). Inhibition of physiologic myocardial FDG uptake in normal rodents: comparison of four pre-scan preparation protocols. Adv Mol Imaging.

[76] Okumura W (2004). Usefulness of fasting 18F-FDG PET in identification of cardiac sarcoidosis. J Nucl Med.

[77] Kreissl MC (2011). Influence of dietary state and insulin on myocardial, skeletal muscle and brain [18F]- fluorodeoxyglucose kinetics in mice. EJNMMI Res.

[78] Thackeray JT, Bankstahl JP, Wang Y, Wollert KC, Bengel FM (2015). Clinically relevant strategies for lowering cardiomyocyte glucose uptake for 18F-FDG imaging of myocardial inflammation in mice. Eur J Nucl Med Mol Imaging.

[79] Laforest R, et al. Measurement of input functions in rodents: challenges and solutions. Nucl Med Biol 2005;32(7):679–685. 10.1016/j.nucmedbio.2005.06.012. 10.1016/j.nucmedbio.2005.06.012 16243642

[80] Meyer M, Le-Bras L, Fernandez P, Zanotti-Fregonara P (2017). Standardized input function for 18F-FDG PET studies in mice: A cautionary study. PLoS ONE.

[81] Amirrashedi M, Zaidi H, Ayer MR. Towards quantitative small-animal imaging on hybrid PET/CT and PET/MRI systems. Clin Transl Imaging 2020;8:243–263. 10.1007/s40336-020-00376-y.

